# The CRE1 Cytokinin Pathway Is Differentially Recruited Depending on *Medicago truncatula* Root Environments and Negatively Regulates Resistance to a Pathogen

**DOI:** 10.1371/journal.pone.0116819

**Published:** 2015-01-06

**Authors:** Carole Laffont, Thomas Rey, Olivier André, Mara Novero, Théophile Kazmierczak, Frédéric Debellé, Paola Bonfante, Christophe Jacquet, Florian Frugier

**Affiliations:** 1 CNRS, Institut des Sciences du Végétal (ISV), avenue de la Terrasse, 91198 Gif-sur-Yvette cedex, France; 2 Université de Toulouse, UPS, Laboratoire de Recherche en Sciences Végétales, BP42617, Auzeville, F-31326, Castanet-Tolosan, France; 3 CNRS, Laboratoire de Recherche en Sciences Végétales, BP42617, Auzeville, F-31326, Castanet-Tolosan, France; 4 Dipartimento di Scienze della Vita e Biologia dei Sistemi, Università degli Studi di Torino, 10125 Torino, Italy; 5 INRA, Laboratoire des Interactions Plantes-Microorganismes (LIPM), UMR441, Castanet-Tolosan, F-31326, France; 6 CNRS, Laboratoire des Interactions Plantes-Microorganismes (LIPM), UMR2594, Castanet-Tolosan, F-31326, France; University of Nottingham, UNITED KINGDOM

## Abstract

Cytokinins are phytohormones that regulate many developmental and environmental responses. The *Medicago truncatula* cytokinin receptor MtCRE1 (Cytokinin Response 1) is required for the nitrogen-fixing symbiosis with rhizobia. As several cytokinin signaling genes are modulated in roots depending on different biotic and abiotic conditions, we assessed potential involvement of this pathway in various root environmental responses. Phenotyping of *cre1* mutant roots infected by the *Gigaspora margarita* arbuscular mycorrhizal (AM) symbiotic fungus, the *Aphanomyces euteiches* root oomycete, or subjected to an abiotic stress (salt), were carried out. Detailed histological analysis and quantification of *cre1* mycorrhized roots did not reveal any detrimental phenotype, suggesting that MtCRE1 does not belong to the ancestral common symbiotic pathway shared by rhizobial and AM symbioses. *cre1* mutants formed an increased number of emerged lateral roots compared to wild-type plants, a phenotype which was also observed under non-stressed conditions. In response to *A. euteiches*, *cre1* mutants showed reduced disease symptoms and an increased plant survival rate, correlated to an enhanced formation of lateral roots, a feature previously linked to *Aphanomyces* resistance. Overall, we showed that the cytokinin CRE1 pathway is not only required for symbiotic nodule organogenesis but also affects both root development and resistance to abiotic and biotic environmental stresses.

## Introduction

Cytokinins control many aspects of plant development as well as response to environmental stresses [1–3]. Considering root system development, exogenous cytokinin application as well as ectopic expression or mutation of cytokinin synthesis/degradation/signaling genes revealed the negative function of these phytohormones in root growth and lateral root formation [4–6]. First, cytokinins affect *Arabidopsis thaliana* vascular bundle patterning, regulating negatively protoxylem differentiation depending on the CRE1/AHK4 (Cytokinin Response 1 / Authentic Histidine Kinase 4) cytokinin receptor [7]. Second, these phytohormones regulate *Arabidopsis* root meristem activity depending on the AHK3 receptor, by enhancing the transition of root meristematic cells towards elongation and differentiation [8]. Third, cytokinins also negatively control *Arabidopsis* lateral root formation, blocking initial pericycle cell divisions and affecting patterning of lateral root primordia [9]. This regulation of lateral root formation involves, depending on studies, combinations of AHK2, AHK3 and/or CRE1/AHK4 cytokinin receptors [10–12]. In legumes, specific RNAi (RNA interference) approaches against three different *Medicago truncatula* cytokinin receptors revealed that the CRE1 pathway is similarly required to inhibit lateral root formation [13]. Cytokinins are also crucial to control the development of another root lateral organ, the nitrogen-fixing nodule, which forms on legume root systems in nitrogen-deprived soils following the interaction with symbiotic bacteria collectively referred to as “Rhizobia” [14]. Indeed, the *Lotus japonicus* or *M. truncatula* homologs of *Arabidopsis* CRE1/AHK4 are required for an efficient nodulation [14, 15]. In addition, a gain of function mutation in the *Lotus* LHK1 (Lotus Histidine Kinase 1) cytokinin receptor, as well as analysis of *CRE1* spatial expression and cytokinin response activation domain in *Medicago*, clearly indicate that the cytokinin-dependent CRE1 pathway is necessary and sufficient to activate cortical cell divisions and nodule organogenesis [16–18]. Most land plants are able to establish another type of endosymbiosis with arbuscular mycorrhizal (AM) fungi [19–20]. Potential roles of phytohormones in these interactions, including cytokinins, are however poorly documented [21].

Cytokinins have also been linked to other environmental responses in addition to symbiosis. Cytokinin content and signaling is differentially modulated by various abiotic stresses depending on the organ considered, and also dynamically depending on length of treatments [1, 22–24]. Besides an early regulation which may be directly involved in the stress response, cytokinins are additionally involved in metabolic, growth, and developmental adaptations, as well as in senescence processes induced by detrimental conditions. Use of transgenic strategies to increase cytokinin degradation or to reduce cytokinin synthesis suggest that these hormones are overall negatively regulating stress response, as these cytokinin deficient plants are salt or drought stress tolerant [1, 24]. Moreover, a specific Histidine Kinase, AHK1, proposed to act as an osmotic sensor, interferes with AHKs involved in cytokinin perception to determine *Arabidopsis* stress tolerance [25]. Cytokinin signaling Histidine Phosphotransfer Protein (HPT) or Response Regulators (RR) mutants also display stress response phenotypes [23, 26, 27]. Accordingly, many Type A RRs (RRAs [28]) and several cytokinin receptors are transcriptionally regulated by abiotic stresses, in *Arabidopsis* or other plants such as the *M. truncatula* legume [29–30].

Cytokinins recently emerged as a regulatory pathway affecting pathogenic plant-microbe interactions [31]. Plant pathogens inducing galls, such as *Agrobacterium tumefaciens, Rodococcus fascians*, or bacterial symbionts of leaf-miners insects forming green islands, use different strategies to modify plant cytokinin response and therefore plant development [32–35]. Recently, a crosstalk was proposed between the salicylic acid defense hormone and cytokinins in the *Pseudomonas syringae /Arabidopsis* interaction. Heterodimerization of the ARR2 cytokinin-related transcription factor with the TGA3 salicylic acid-response factor enhanced transcriptional activation of the *Pathogenesis Related protein 1 (PR1)* target gene [36]. As ARR2 is also involved in controlling leaf senescence as well as different developmental processes [37], this suggests that cytokinins may directly interfere with defense response but also indirectly through their effects on plant development, metabolism and physiology.

In this work, we determined the implication of cytokinin pathways depending on the MtCRE1 receptor, essential for nodule organogenesis, in several *M. truncatula* root biotic and abiotic responses, highlighting the interaction of these environmental factors with the regulation of root development and root system architecture.

## Materials and Methods

### Plant material and growth conditions

Seeds of *M. truncatula cre1* mutants (*cre1-1* and *cre1-2* alleles; [18]) and their respective Wild-Type (WT) sibling controls were scarified in sulfuric acid during 3 min, rinsed four times with distilled water and surface sterilized for 20 min in bleach (12% [v/v] sodium hypochlorite). After washing with sterilized water, seeds were sown on 1% water-agar plates and stratified for three days at 4°C before incubating overnight at 24°C in the dark. Germinated seedlings were transferred to square plates containing appropriate medium (see below) and grown vertically in a growth chamber at 24°C under long-day conditions (16 h light at 150 μE intensity).

For each experiment, two to three independent biological replicates were performed. A non-parametric Kruskal-Wallis test (available in the Xlstat software; http://www.xlstat.com/) was used to assess significant differences.

### Arbuscular mycorrhiza and *Aphanomyces* infection and phenotyping

For arbuscular mycorrhiza establishment, sterilized seeds were pre-germinated in Petri dishes filled with agar/water (0.6%). Root systems were placed between two Millipore membranes along with 15–20 *Gigaspora margarita* (strain BEG 34) spores previously sterilized with Chloramine T (3% w/v [weight/volume], Sigma) and Streptomycine sulphate (0.3% w/v, Sigma). After four weeks of co-culture in a Magenta box (about 20 cm in height), plants can develop a very long root system (around 1 m of cumulated length). These roots were sampled and stained with cotton blue (0.1% w/v in lactic acid [38]) to detect the presence of intraradical fungal structures. For each genotype, 400 root segments of 1 cm long, belonging to four independent plants, were observed using a Zeiss Primo Star optical microscope. Pictures were taken with a Leica DFC425 C camera.

For *Aphanomyces euteiches*, zoospores of the strain ATCC 201684, a pea isolate, were produced as described by [39] and adjusted to 10^5^ spores/ml. Seedlings were inoculated one day after transfer onto M medium [40] with a 5 μl droplet of spore suspension deposited in the middle of the root. To assess mutant resistance, three independent *in vitro* infection assays were performed with 10 to 20 inoculated plants/genotype in each repeat. Relative length of tissues displaying symptoms (14 days post-inoculation, or dpi), along with percentages of dead plants (21 dpi), which have been shown to be the most significant parameters to assess the level of resistance of *M. truncatula* to *A. euteiches*, were recorded, as described by [41]. Symptoms were quantified with the ImagePro image analysis software. The same growth conditions were used to produce material for RNA extraction and subsequent real time RT-PCR analyses, except that plants were grown *in vitro* ten days before root inoculation with *A. euteiches* zoospores.

### Salt treatments and phenotyping

For real-time RT-PCR experiments, fifteen germinated seedlings were placed on a grid in a Magenta box with 30 ml of low-nitrogen ‘i’ liquid medium [13] and grown in a shaking incubator (125 g) at 24°C under long-day conditions (16 h light/8 h dark). After five days, seedlings were treated with or without 100 mM NaCl and maintained under the same growth conditions for various incubation times (0, 1 and 3 h). These conditions allow having homogeneous root responses when short-term salt treatments are used. Roots were collected at the indicated time points and immediately frozen in liquid nitrogen for RNA extraction.

To monitor the effect of salt stress on root growth over several days, we used *in vitro* agar conditions. Germinated seedlings were placed on growth papers (Mega International, http://www.mega-international.com/index.htm) on ‘i’ medium. After three days, papers were transferred on a fresh ‘i’ medium supplemented or not with 100 mM NaCl. Position of root tips was marked at the time of transfer, and root growth was measured from this point seven days after, using the ImageJ software (http://rsbweb.nih.gov/ij/).

### Gene expression analysis

Affymetrix microarray data available on the *M. truncatula* Gene Expression Atlas (MtGEA; http://mtgea.noble.org/v3/) were used to analyze expression of cytokinin signaling genes: Histidine Kinases (HKs), Histidine Phosphotransfer proteins (HPs), and Response Regulators (RRs). Correspondence between genome (v.3.5.1) and Affymetrix IDs was determined using the “Nickname” tool from the “Legoo” website (http://www.legoo.org/). Hierarchical clustering was performed on logarithmic gene expression ratio between the different conditions and their respective controls, based on Euclidean distance and average gene clustering using the MeV software (http://www.tm4.org/mev/).

For real time RT-PCR, total RNA was extracted from frozen roots using the RNeasy plant mini kit (Qiagen, http://www.qiagen.com/). First-strand cDNA was synthesized from 1.5 μg of total RNA using the Superscript II first strand synthesis system (Invitrogen, www.invitrogen.com/). Primer design was performed using Primer3 software (http://frodo.wi.mit.edu/cgi-bin/primer3/). Primer combinations showing a minimum amplification efficiency of 90% were used in real-time RT-PCR experiments (S1 Table). Real-time RT-PCR reactions were performed using the Light Cycler Fast Start DNA Master SYBR Green I kit on a Light Cycler apparatus according to manufacturer’s instructions (Roche, http://www.roche.com/). Cycling conditions were as follows: 95C for 10 min, 40 cycles at 95C for 15 sec, 60C for 15 sec, and 72C for 15 sec. PCR amplification specificity was verified using a dissociation curve (55–95C). A negative control without cDNA template was always included for each primer combination. Technical replicates (on two independent syntheses of cDNA derived from the same RNA sample) and two independent biological experiments were performed. Ratios were done with constitutive controls for gene expression to normalize the data between different biological conditions. *MtRBP1* and *MtH3L* were selected using the Genorm software (http://medgen.ugent.be/~jvdesomp/genorm/ [42]) as reference genes (primers shown in S1 Table), and the value of the experimental control condition was set up to 1 as a reference to determine fold changes.

### Histological analyses of root meristem and vasculature

To analyze vasculature patterning, seven-day-old seedlings grown *in vitro* on low-nitrogen liquid medium (‘i’ [13]) were used. Roots segments (approximately 10 mm) were cut at 15 or 40 mm from the root tip and fixed in 2% glutaraldehyde (Sigma). Roots were embedded in 3% agarose and then sliced into 80 μm transversal sections using a VT 1200S vibratome (Leica Microsystems, http://www.leica-microsystems.com/). Vibratome sections were then mounted in water and observed either in bright field or using 340–380 nm excitation/450–490 nm emission filters (to detect the blue autofluorescence of endodermis and metaxylem) on a Reichert Polyvar microscope equipped with a Retiga 2000 Camera (QImaging, www.qimaging.com/).

To analyze root meristem patterning and size, a “PI-PA staining” derived from [43] was used. Root tips (10 mm) were fixed in 50% methanol/ 10% acetic acid at 4°C for 1 h, rinsed twice with distilled water before incubation in a bleach solution (sodium hypochlorite 7.5% active chloride for 20 min at room temperature). Tips were rinsed again twice with distilled water and incubated in 0.1% triton for 10 min, and then in 1% periodic acid (PA; Sigma) for 30 min. After two washings with distilled water, root tips were transferred in Schiff reagent with propidium iodide (100 mM sodium metabisulphite [Sigma] / 0.15 N HCl / propidium iodide [PI; Sigma], freshly added to a final concentration of 100 μg/mL) for at least 2 h. Samples were then transferred onto slides and mounted in a clearing solution (4 g chloral hydrate, 1 mL glycerol, and 2 mL water; Sigma). Stained root tips were analyzed on an inverted confocal scanning microscope (Leica Microsystems), using a 488 nm excitation wavelength, and fluorescence emission was collected between 520 and 720 nm. Series of confocal images were merged using the Photomerge macro from Photoshop (Adobe). Measurements of the meristematic zone (from the quiescent center to the first elongating outer cortical cell; S1 Fig.) and elongation zone (from the first elongating cell to the first emerged root hair, corresponding to a fully elongated outer cortical cell; S1 Fig.) were done using ImageJ.

### Nitrogen fixation activity

To measure nitrogenase activity of symbiotic nodules, an Acetylene Reduction Assay (ARA) was performed on individual plants with a protocol that was derived from [44]. Five weeks after inoculation with *Rhizobium*, the plants were placed into 10 ml glass vials that were sealed with rubber septa. Acetylene was injected into each vial, and after 1 h of incubation at room temperature, the produced ethylene was measured using Gas Chromatography (7820A, Agilent technologies).

## Results

### Transcriptional regulation of cytokinin metabolic and signaling pathways in response to various environmental conditions

**Figure 1:**
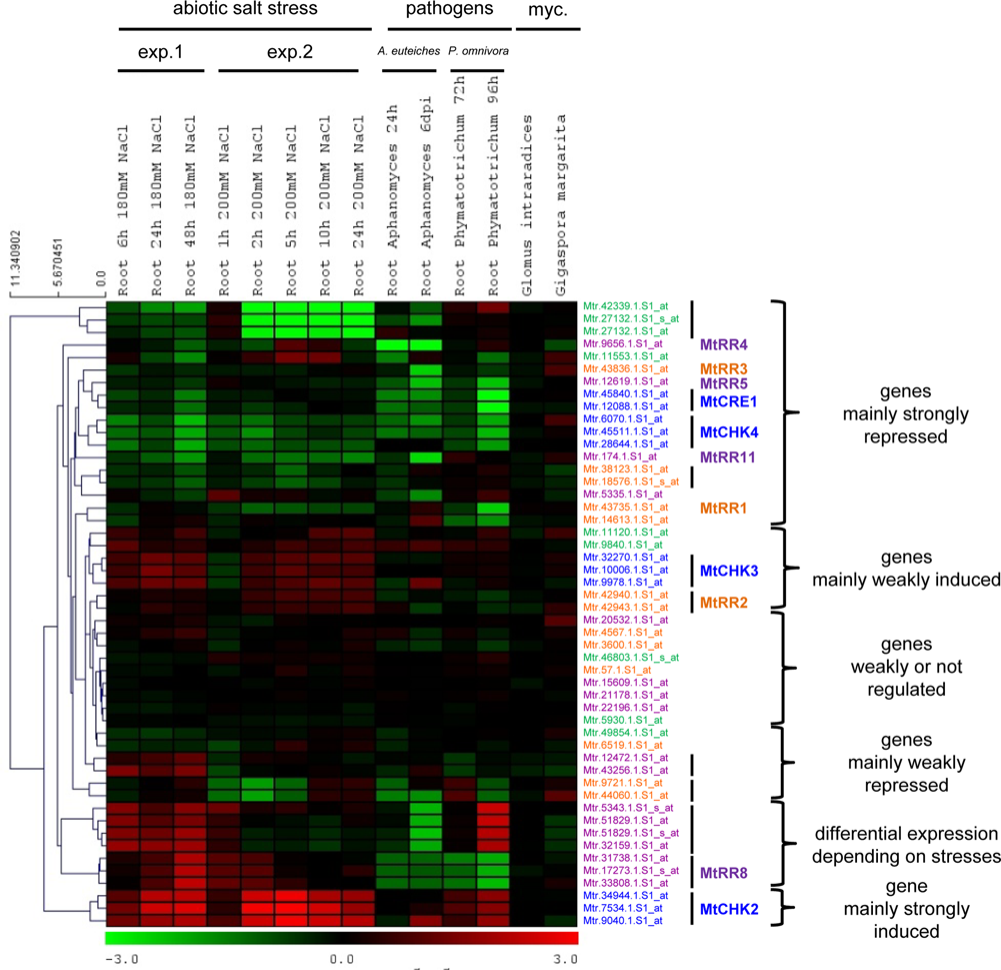
Heat map of *Medicago truncatula* cytokinin signaling gene expression in roots exposed to various environmental conditions. Selected Affymetrix array data corresponding to roots under various abiotic and biotic conditions were retrieved from the *M. truncatula* Gene Expression Atlas (MtGEA) database: “myc.”, mycorrhized roots [70, 71]; salt stress, two independent experiments (exp. 1 and exp. 2 [72]); *Phymatotrichopsis omnivora* and *Aphanomyces euteiches* pathogens (respectively [68, 73]). All probes corresponding to cytokinin signaling genes were included in the heat map, which was constructed with logarithmic gene expression ratio between the different conditions and their respective controls, based on Euclidean distance and average clustering probes across the experimental conditions included, using the MeV software. Color scale ranges from eight time fold-repression in green (log2 = −3) to eight time fold-induction in red (log2 = 3). Accession numbers correspond to Affymetrix probes (correspondence with gene ID in S2 Table), and multiple probes corresponding to a single gene are indicated by a vertical black bar on the right. Colors indicate cytokinin signaling gene families: in blue, CHKs (CHASE domain containing Histidine Kinases); in green, HPTs (Histidine PhosphoTranfert proteins); in orange, RRBs (Type-B Response Regulators); in violet, RRAs (Type-A Response Regulators). MtCRE1, MtCHK2/HK2, MtCHK3/HK3, MtRR1 to MtRR5 gene names were defined in [13]; MtRR8 in [46]; MtRR11 in [45]. For other IDs, no gene name is available in the literature.

We previously documented that in the *M. truncatula* legume, cytokinin signaling genes, and notably Response Regulators (RRs), were transcriptionally regulated in response to symbiotic rhizobia inoculation and salt stress [13, 29]. We therefore analysed transcriptional regulations of all *M. truncatula* cytokinin signaling (CHKs, HPTs, and RRBs and RRAs [28]; S2 Table) and metabolic (biosynthesis: Iso-Pentenyl Transferases, IPTs, and a “Lonely Guy” cytokinin activating enzyme, LOG; degradation: Cytokinin Oxidases/ Deshydrogenases, CKX; S2 Table) genes for which probes were available on Affymetrix microarrays. Expression of all these genes was analysed in response to various environmental cues affecting the root system, including the mycorrhizal symbiotic fungi *Glomus intraradices* (now *Rhizophagus irregularis*) and *Gigaspora margarita*; an abiotic stress, salt; and root pathogens, the biotrophic oomycete *Aphanomyces euteiches* and the necrotrophic fungus *Phymatotrichopsis omnivora* (Fig. 1 for cytokinin signaling genes and S2 Fig. for metabolic genes).

Global analysis of *M. truncatula* cytokinin metabolic genes revealed a tendency for *IPT* cytokinin biosynthetic genes to be downregulated by abiotic and biotic stresses, even though usually weakly, whereas *CKX* genes were upregulated (S2 Fig.). Cytokinin signaling gene transcriptional regulations revealed six main clusters: (1) genes mainly repressed in the various conditions analysed (18 probes corresponding to 12 genes); (2) genes mainly weakly repressed (four probes corresponding to three genes); (3) genes weakly or not regulated across the various selected conditions (nine probes corresponding to nine genes); (4) genes mainly weakly induced (nine probes corresponding to five genes); (5) genes showing a differential expression between the different stresses (seven probes corresponding to two *RRA* genes); and (6) one gene mainly strongly induced (three probes corresponding to Mt*CHK2*; Fig. 1). Some of these co-regulated gene clusters include members of CHKs, HPTs, RRBs and RRAs families, and may then define specific phosphotransfer pathways.

Considering the biological conditions analysed, and in contrast to symbiotic nodulation, no strong regulation of *Medicago* cytokinin signaling and metabolic genes was observed in response to the AM fungi, independently of the gene family considered (Figs. 1 and S2). Salt stress response analyses, based on two independent experiments (NaCl 200 mM for 1 to 24 hours and NaCl 180 mM for 6 to 48 hours), revealed that a specific *CKX* gene (out of four; corresponding to probes Mtr.38799.1.S1_s_at and Mtr.44219.1.S1_at) is strongly up-regulated whereas two *IPT* genes (out of three; corresponding to probes Mtr.12113.1.S1_at and Mtr.48824.1.S1_at) were down-regulated (S2 Fig.). Concerning cytokinin signaling genes (Fig. 1), among the four putative cytokinin receptors present in the *M. truncatula* genome, two are salt stress-induced (MtCHK2 and MtCHK3, closely related to AHK3 in Arabidopsis), whereas the two others *CHK*s are repressed (MtCRE1/CHK1 and MtCHK4, respectively closely related to Arabidopsis AHK4/CRE1 and AHK2). Two *HPT* genes (out of seven; corresponding to probes Mtr.42339.1.S1_at / Mtr.27132.1.S1_s_at / Mtr.27132.1.S1_at and Mtr.11553.1.S1_at) are strongly repressed by the stress conditions, whereas the five other genes are not or weakly regulated. At least five specific *RRB*s (including Mt*RR1* and Mt*RR3*; [18]) and four *RRA*s (including Mt*RR4*, Mt*RR5* and Mt*RR11* [18, 45]) are salt-repressed, whereas at least one *RRB* (corresponding to probes Mtr.9721.1.S1_at and Mtr.44060.1.S1_at) and three *RRA*s (including Mt*RR8* [46]) are induced. During the root pathogenic interactions analysed (*A. euteiches* 1 and 6 dpi and *P. omnivora* 72 and 96 hpi), most of cytokinin signaling genes were repressed, including two of the *CHK*s, Mt*CRE1* and Mt*CHK4*. Only two genes were strongly upregulated: Mt*CHK2* by the two pathogens, and a *RRA* (corresponding to probes Mtr.5343.1.S1_s_at, Mtr.51829.1.S1_at, Mtr.51829.1.S1_s_at and Mtr.32159.1.S1_at) differentially expressed depending on the pathogen used (repression by *Aphanomyces* and induction by *Phymatrotrichopsis* for the latest time point). Finally, among all cytokinin genes analysed, a single *RRA* gene (Mt*RR8*) showed a clearly opposite regulation between salt stress (induced) and biotic stresses (repressed).

As various abiotic and biotic stress conditions widely affect expression of cytokinin pathways in *M. truncatula* roots in a complex pattern, we next wanted to determine whether these putative connections between cytokinins and plant responses to different environmental factors could be validated using loss of function mutants affecting CRE1/CHK1 [18].

### *cre1* mutants do not display any defect in their response to the arbuscular mycorrhizal fungus *Gigaspora margarita*

**Figure 2:**
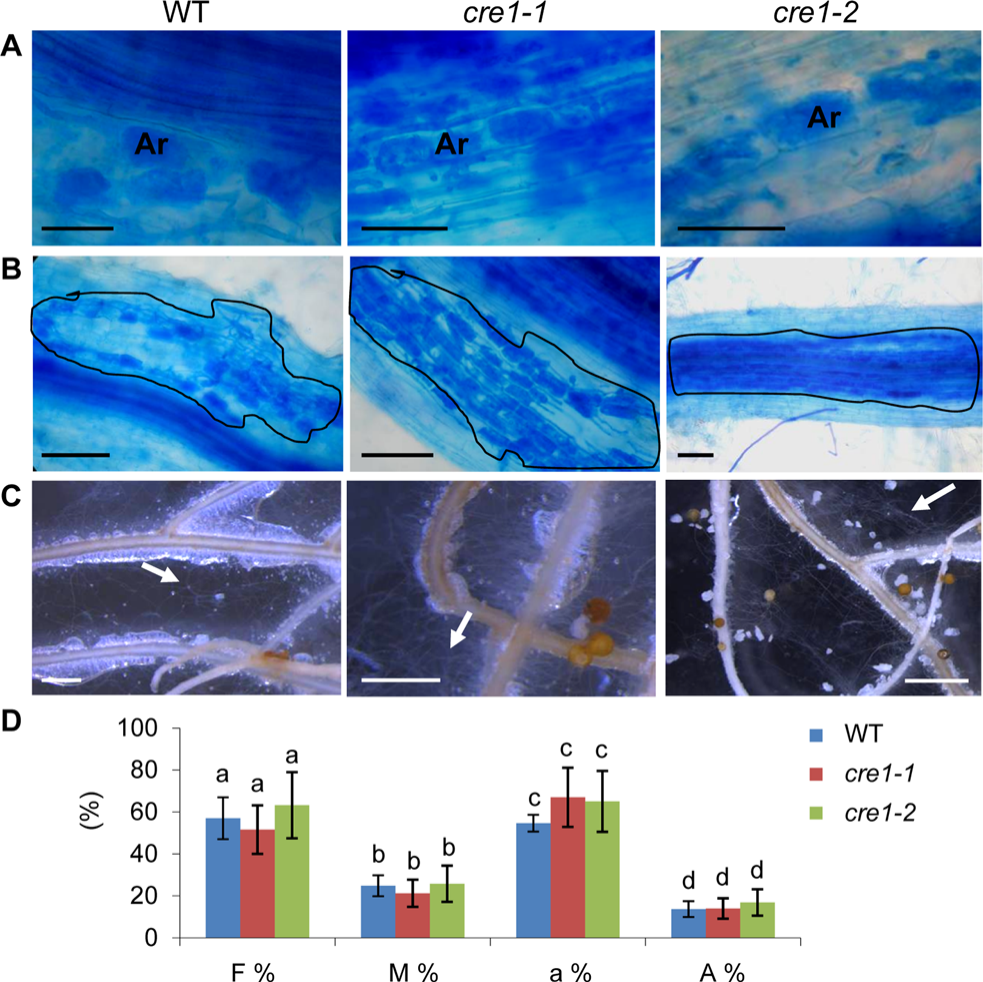
Mycorrhizal colonization of *cre1* mutants is similar to wild-type. **A–C**. Representative pictures of the Wild-Type (WT) and of the *cre1* mutant phenotype (*cre1-1* and *cre1-2* alleles) after mycorrhizal inoculation: **A**, Arbuscule (Ar) development in root cortical cells; **B**, intraradical colonization (lines indicate the colonized regions); **C**, Extraradical mycelium development (arrows indicate external mycorrhizal hyphae). Bars correspond to 50 μm in A, 100 μm in B, and 2 cm in C. **D.** Quantification of the mycorrhizal colonization rate of the Wild-Type (WT) and of the *cre1* mutant (*cre1-1* and *cre1-2* alleles) following criteria defined by [47]: F%, frequency of root mycorrhization; M%, intensity of mycorrhization in the root cortex; a%, percentage of arbuscules within infected areas; A%, percentage of arbuscules in the whole root system. Error bars represent standard deviations (n = 4 complete root apparatus analyzed), and a Kruskal-Wallis test was performed to assess significant differences, shown by letters (α<0.05).

As the MtCRE1 cytokinin signaling pathway is crucial for the interaction with the symbiotic bacteria *Sinorhizobium* (now *Ensifer*) *meliloti* [18], we were first interested to determine whether this pathway could also be recruited by the plant to establish symbiotic interaction with the AM fungus *G. margarita*, even though no consistent transcriptional regulation of cytokinin related genes was previously identified (Fig. 1). Wild-Type (WT) or *cre1* mutant genotypes both allowed infection by *G. margarita* in the root epidermis and in cortical cells, as well as arbuscule differentiation (Fig. 2A and B, respectively). In addition, extraradical mycelium development was similar between the different genotypes (Fig. 2C). To evaluate more precisely and quantitatively intraradical colonization and arbuscule differentiation, a cumulated root length of 400 cm (see Methods) was analyzed for each genotype and four parameters were evaluated, based on the protocol set up by [47]: F% (frequency of mycorrhization) indicating the percentage of segments showing internal colonization; M% (intensity of mycorrhization) indicating the average colonization of root segments; a% (percentage of arbuscules in the infected area) quantifying the presence of arbuscules within the infected areas; A% (percentage of arbuscules in the root system) quantifying the presence of arbuscules in the whole root system (Fig. 2D). None of these parameters was able to significantly discriminate *cre1* genotypes from the WT (Kruskal-Wallis test, α<0.05). Overall, the absence of any detectable mycorrhizal phenotype in *cre1* mutants is consistent with the limited transcriptional regulations of cytokinin signaling genes in roots with or without AM fungi.

### *cre1* mutants form an increased number of emerged lateral roots including when challenged by an osmotic stress

**Figure 3:**
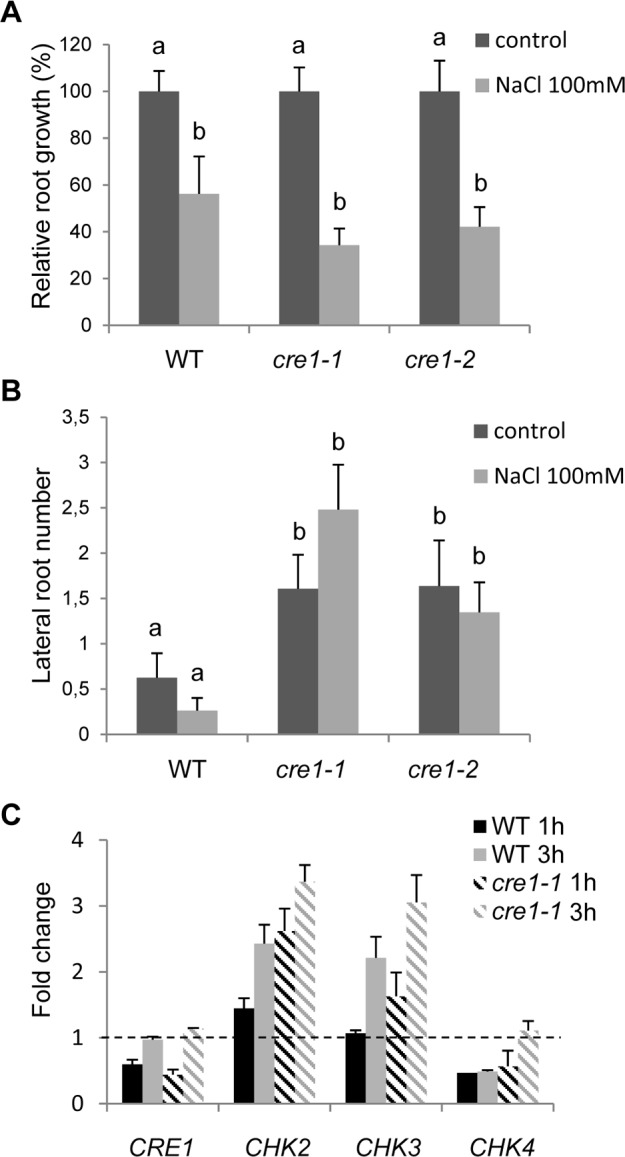
*cre1* mutants form an increased number of lateral roots under control and salt stress conditions. **A.** Root length in the Wild-Type (WT) and in the *cre1* mutant (*cre1-1* and *cre1-2* alleles) was quantified after 7 days on a nitrogen-starved medium (“i” [13]) containing salt (100mM NaCl). **B.** Lateral root number in the Wild-Type (WT) and in the *cre1* mutant (*cre1-1* and *cre1-2* alleles) was determined 7 days after salt treatment (100mM NaCl). In A and B, error bars represent standard deviation (n>20) and a Kruskal-Wallis test was used to assess significant differences (shown by letters, α<0.05). **C.** Real time RT-PCR of putative cytokinin receptors expression in roots of the Wild-Type (WT) and of the *cre1-1* mutant treated by salt (NaCl 100 mM) for 1 or 3 hours. Genes of interest were normalized with the reference gene Mt*RBP1* and calibrated with the non-treated condition (the broken line indicate a ratio of 1). Error bars represent standard deviation of two biological replicates.

To analyze the root responses of *cre1* mutants to an osmotic stress, we used a NaCl concentration previously shown to slightly but significantly inhibit *M. truncatula* Jemalong A17 root growth (NaCl 100 mM [48–51]). No significant difference in the root growth inhibition induced by the salt treatment could be observed between *cre1* and the WT (Fig. 3A; Kruskal-Wallis test, α<0.05). When analyzing the number of emerged lateral roots formed in the different genotypes under salt conditions, an increased number of lateral roots was observed in *cre1* compared to the WT (Kruskal-Wallis test, α<0.05; Fig. 3B). A similar increase in the number of emerged lateral roots was also observed in the *cre1* mutant in the absence of stress (Fig. 3B; Kruskal-Wallis test, α<0.05). As transcriptomic analyses revealed that the three other *CHK*s genes were also regulated in response to salt stress (Fig. 1), we tested their regulation in the *cre1* mutant (Fig. 3C). Salt-regulation of all *CHK*s was maintained in mutants similarly as in the WT. These results suggest that, in addition to the increased ability of *cre1* mutants to form lateral roots, a functional redundancy may exist concerning root growth inhibition by salt, notably between MtCRE1 and MtCHK4, the other salt-repressed CHK.

### *cre1* roots have an altered vascular pole differentiation

**Figure 4:**
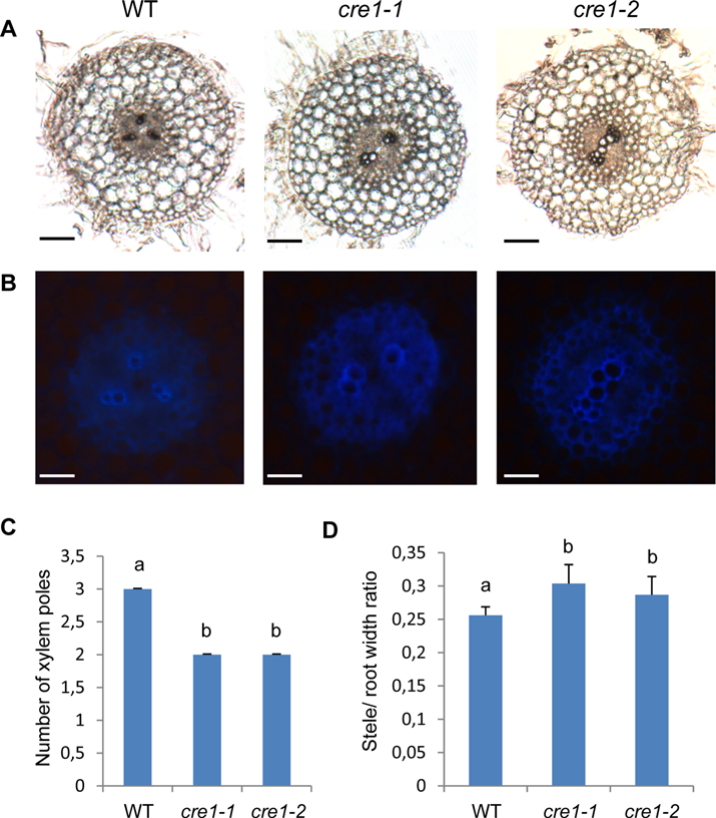
*cre1* mutants show defects in vascular bundle differentiation. **A-B**. Transversal sections of 7 day-old roots in the Wild-Type (WT) and in the *cre1* mutant (*cre1-1* and *cre1-2* alleles). Sections (80 μm) were made at a 1 cm distance from the root tip. **A**, bright field images; **B**, images obtained under UV excitation, revealing the autofluorescence of xylem poles. Bars = 50 μm. **C.** Number of xylem poles in the Wild-Type (WT) and in the *cre1* mutant (*cre1-1* and *cre1-2* alleles) quantified based on previous sections. **D.** Stele/root width ratio in the Wild-Type (WT) and in the *cre1* mutant (*cre1-1* and *cre1-2* alleles) quantified based on previous sections. In C and D, error bars represent standard deviation and a Kruskal-Wallis test was used to assess significant differences (letters, α<0.05; n>6).

As *cre1* mutants showed intrinsic defects in the regulation of lateral root formation independently of the stress condition, we analyzed more in depth potential roles of the *M. truncatula* CRE1 pathway in root development. No difference between meristematic and elongation zone length could be detected between *cre1* mutants and WT roots, nor any defect in root cell-type patterning (i.e. number of cell files; S1 Fig.). This indicates that CRE1 has no major function on its own in the root apical meristem. As lateral root initiation depends on pericycle cell divisions in front of protoxylem poles [5], we wondered whether, in the *cre1* mutant generating more lateral roots, the number of vascular bundles could be affected. Surprisingly, we observed that *cre1* mutants differentiate only two xylem and phloem poles in young root apical regions, whereas WT plants formed three of each poles (Kruskal-Wallis test, α<0.05; Fig. 4A, B and C). In the mutant, a metaxylem diarch structure was then observed in the root apical region. This vascular defect was associated with a slightly wider stele diameter, whereas the overall root diameter remained similar between genotypes (Kruskal-Wallis test, α<0.05; Fig. 4D). This vasculature phenotype was suggestive of a developmental delay, as in more basal root regions, four vascular poles were similarly retrieved in *cre1* mutants and WT roots. This result indicates that CRE1 participates in root vascular bundle differentiation, even though this defect likely does not explain the higher ability of *cre1* to form lateral roots.

### The increased resistance of *cre1* mutants to a root pathogen is correlated with its higher capacity to form lateral roots

**Figure 5:**
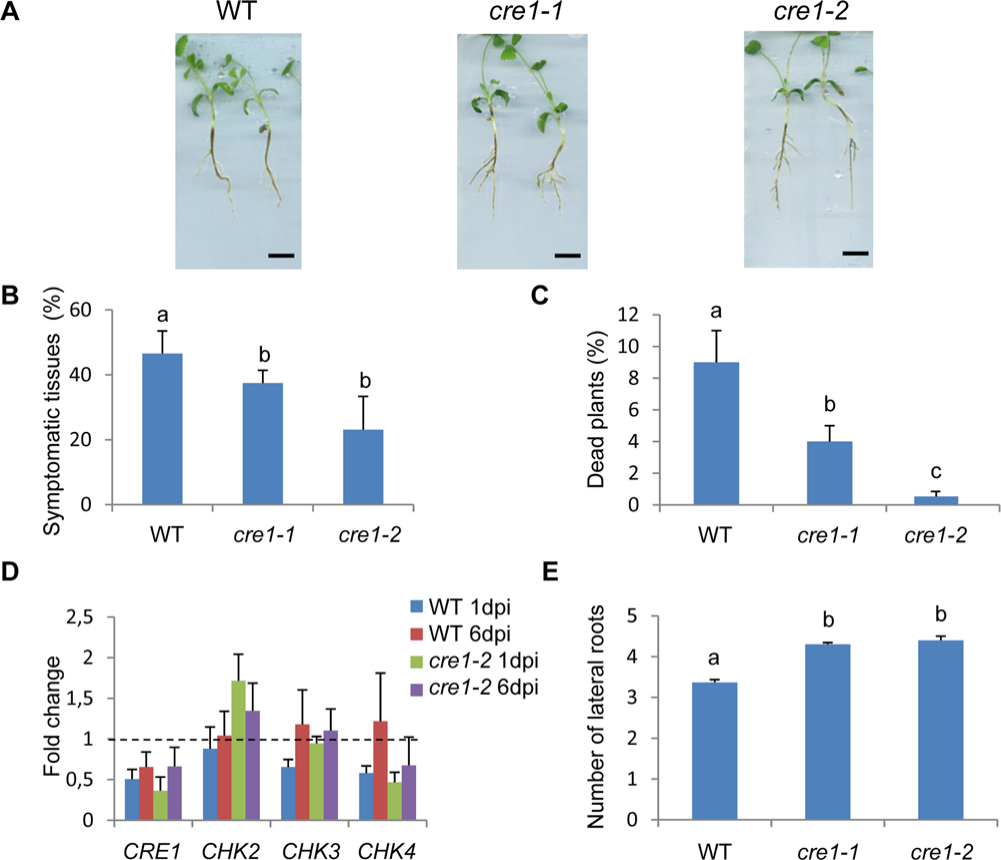
*cre1* mutants resistance to *Aphanomyces euteiches* infection correlates with an increased lateral root formation. **A.** Representative phenotypes of the Wild-Type (WT) and of the *cre1* mutant (*cre1-1* and *cre1-2* alleles) observed 21 days post inoculation (dpi) with *A. euteiches*. **B.** Percentage of symptomatic tissues observed at 14 dpi in the Wild-Type (WT) and in the *cre1* mutant (*cre1-1* and *cre1-2* alleles). **C.** Percentage of dead plants at 21 dpi in the Wild-Type (WT) and in the *cre1* mutant (*cre1-1* and *cre1-2* alleles). **D.** Real time RT-PCR of putative cytokinin receptors expression (*CHK*s) in roots of the Wild-Type (WT) and of the *cre1* mutant (*cre1-2* allele), treated with *A. euteiches* for 1 or 6 days (dpi, days post infection). Genes of interest were normalized with the reference gene Mt*RBP1* and calibrated with the non-treated condition (the broken line indicates a ratio of 1). Error bars represent standard deviation of three biological replicates. **E.** Number of lateral roots emerged at 21 dpi in the Wild-Type (WT) and in the *cre1* mutant (*cre1-1* and *cre1-2* alleles). In B, C and E, error bars represent confidence interval (α = 0.05; n>30) and a Kruskal-Wallis test was used (shown by letters, α<0.05).

To analyze whether the MtCRE1-dependent cytokinin pathway could be involved in response to a biotic stress, we challenged *cre1* plants with *Aphanomyces euteiches,* a root oomycete. The *M. truncatula* Jemalong A17 genotype in which *cre1* mutants were generated was previously reported to confer a partial quantitative resistance to this pathogen [41]: *A. euteiches* induces root symptoms and can accomplish a full biological cycle in the root cortical cells, but the plant can still grow due to the activation of several defense mechanisms that prevent the parasite to invade the root stele. Propagation of *A. euteiches* throughout the root and finally inside stem tissues can be recorded by monitoring length of brown tissues [41, 52]. Previous quantitative parameters developed to assess the level of plant resistance were used to phenotype *cre1* mutants upon *A. euteiches* infection. This includes notably the percentages of symptomatic brown tissues observed 14 days post-inoculation (dpi; Fig. 5A and B) on roots and sometimes on hypocotyls, and the percentage of dead plants (Fig. 5C). A significant decrease in symptomatic tissues was observed for both *cre1* alleles, along with an improvement of plant survival to the pathogen (Fig. 5A–C; Kruskal-Wallis test, α<0.05). As Mt*CHK4* and Mt*CRE1* were similarly downregulated by biotic stress conditions (Fig. 1), we analyzed expression of *CHK*s in the *cre1* mutant in response to *Aphanomyces* (6 dpi; Fig. 5D). No major difference between the expression of the four *CHK*s tested was identified between *cre1* and WT roots (Fig. 3C), as previously observed for the salt stress response. Interestingly, the *cre1* resistance phenotype was correlated with the increased ability of this genotype to form lateral roots (Fig. 5A and E; Kruskal-Wallis test, α<0.05), as previously observed in response to salt stress (Fig. 3B). Overall, these results indicate that MtCRE1 is a negative regulator of lateral root formation and of plant immunity in response to *A. euteiches.*

## Discussion

As MtCRE1 is required for symbiotic nodule formation [18], we wanted to determine if this pathway was also involved in the mycorrhizal symbiotic interaction. Careful qualitative and quantitative analyses of fungal infection, colonization and arbuscules differentiation failed to reveal any significant defect between *cre1* and WT roots. Together with the limited transcriptional regulation of cytokinin-related genes, our results indicate that the central and unique role of cytokinins and MtCRE1 in nodulation is not shared during the mycorrhizal interaction. In contrast to many early nodulation genes identified, the MtCRE1 cytokinin receptor therefore does not belong to the ancestral common symbiotic (SYM) pathway shared between rhizobial and AM symbioses [20, 53, 54]. However, we cannot rule out that other cytokinin receptor(s) may act redundantly to control mycorrhizal infection, despite no current data convincingly points for a role of cytokinins in this symbiotic interaction [21]. RNAi experiments previously showed that only MtCRE1 has a major role in nodulation [13], and similarly amongst the different *Lotus japonicus* cytokinin receptors, only the CRE1 functional homolog, LHK1, has a major symbiotic function [15, 17]. This differential role of cytokinins between the two endosymbioses suggests that MtCRE1 is not involved in a process evolutionary conserved, such as LipoChitoOligosaccharidic (LCO) signaling (Myc and Nod LCOs [54]) or symbiont infection progression. In contrast, MtCRE1 is likely required for a process specifically associated with early stages of nodulation and which does not occur in the mycorrhizal interaction, such as the reactivation of cortical cell divisions leading to nodule organogenesis. Accordingly, the gain of function LHK1 mutant *snf2* forms nodules spontaneously, independently of any symbiont recognition or infection [17].

Analysis of the *cre1* mutant root response to various environmental conditions was the opportunity to uncover additional functions of this pathway in root development. The increased ability of the *cre1* mutant to form lateral roots was consistent with previous results obtained using a RNAi strategy [13]. As AM fungi preferentially colonize lateral roots [55] and as the AM interaction is not defective in *cre1* roots (this study), the increased ability of the mutant to form lateral roots may then even allow an enhanced mycorrhizal colonization. Many studies additionally demonstrated that the AM colonization affects root system architecture, and generally promotes lateral root formation [55]. The underlying mechanisms are however still not fully understood. Two main non-exclusive explanations are currently proposed: lateral root formation would be induced thanks to the improved nutrient acquisition mediated by the symbiotic AM fungi [56]; and as a direct symbiotic response involving perception and signalling of fungal molecules [57].

In addition to interfering with lateral root formation, detailed histological analysis of *cre1* root apices revealed a vasculature patterning defect in the differentiation zone, even though in more mature regions a WT pattern was recovered. This phenotype however does not easily explain the enhanced ability of *cre1* mutants to form lateral roots, and may in contrast even rather limit its occurrence. Interestingly, high levels of cytokinins detected in *R50* pea mutants are correlated with an increased number of root vascular poles [58]. These results suggest that the role of CRE1 in vascular development seems conserved between *Arabidopsis* and *Medicago* [7], even though the transient phenotype identified suggests a functional redundancy with other CHKs.

Beside controlling many aspects of plant development, including root and symbiotic nodule organogenesis, cytokinins were recently linked to an increased number of environmental responses, such as nutrient availability or abiotic and biotic stresses [2, 3, 59]. Indeed, some connections between cytokinin regulation and plant responses to various abiotic stresses have been revealed by transcriptomic analyses [1, 22–24]. However, depending on each member of the gene families considered, on stress treatments, and on plant organs, opposite regulations are observed, indicating that the output of the crosstalk between abiotic stress and cytokinin response is difficult to predict based on transcriptional regulations [2]. In *M. truncatula* also, connections between cytokinin signaling and stress were previously highlighted [29]. Whereas the present study was performed in the Jemalong A17 genotype, this previous analysis was carried out in the R108 genotype, considered as a subspecies of *M. truncatula* (*tricycle* [60]). Interestingly, Mt*CRE1* shows an opposite salt regulation in these two genotypes that may be correlated to their different salt-sensitivity [61]. The meta-analysis performed in the present study revealed an increased complexity of these regulations, with many individual genes showing a highly contrasted expression pattern inside each gene family (eg CHKs, HPTs, RRAs and RRBs). Most of cytokinin-related genes have however a similar trend of regulation between abiotic and biotic stress conditions, and only a few genes (such as Mt*RR8*) showed a clear-cut antagonistic regulation depending on the nature of the stress. In addition to MtCRE1, other CHKs are transcriptionally regulated in response to salt or *Aphanomyces*, suggesting that different CHKs may play a role in the resistance/sensitivity to these environmental conditions. In *cre1* mutant roots, we showed that *CHK* transcriptional regulations by salt stress were maintained, indicating indeed that potential redundant functions may exist. Interestingly, in contrast to *Arabidopsis* where expression of all cytokinin receptors is induced by abiotic stresses [25], Mt*CHK2* and Mt*CHK3* (closely related to the *Arabidopsis* AHK3 cytokinin receptor) are induced by salt, whereas Mt*CRE1* and Mt*CHK4* genes (respectively closely related to AHK4/CRE1 and AHK2 in *Arabidopsis*) are negatively regulated by salt stress. Likewise for *Medicago* roots challenged with pathogens, Mt*CHK2* is upregulated whereas Mt*CRE1* and Mt*CHK4* are downregulated. This suggests that complex interactions between stress and CHK-dependent cytokinin pathways occur in *Medicago* roots. Identification of other *chk* mutants and generation of multiple mutants affecting predicted functionally redundant genes, based on their expression pattern (such as *CRE1* and *CHK4*), are needed to have a better understanding of these crosstalks.

Our study shows that roots of the *cre1* cytokinin receptor mutants are more resistant to the *A. euteiches* oomycete. In *Arabidopsis,* the *ahk3/ahk4* cytokinin receptor mutant is resistant to the actinomycete *Rhodococcus fascians* [62], indicating that, as observed in our study, cytokinin signaling pathways reduce the level of plant immunity and/or promote root microbe pathogenicity and pathogen development. Accordingly, a recent report showed that the *Medicago cre1* mutant was also more resistant to *Ralstonia solanacearum* pathogenic root bacteria [63]. In contrast, inoculated leaves of the three *Arabidopsis ahk* mutants displayed an enhanced susceptibility to the *Hyaloperonospora arabidopsidis* oomycete [30]. Depending on the pathogenic interactions considered, cytokinins can then promote either plant defense or pathogenicity (this study and [30, 36, 62]). In addition to specificities linked to the plant / pathogen used, these apparently conflicting functions of cytokinins, promoting either pathogen tolerance or susceptibility, may also depend on the organ considered. Such differential organ-specific effects are likely related to the multiple functions of cytokinins, which in addition to directly mediating pathogenic responses, also indirectly affect pathogen growth and plant defense through their effects on development. Interestingly, the increased *Aphanomyces* resistance level observed in *Medicago* roots is correlated with the ability of this mutant to generate more lateral roots (this study and [41, 64]) a developmental process known to be negatively regulated by cytokinins [5, 9]. Truong et al. [65] recently showed that the *M. truncatula* B9 mutant developing a reduced number of lateral roots was hypersensitive to *Aphanomyces*, even though no direct measurement of lateral root formation in response to inoculation was provided. In addition, a recent GWAS analysis revealed that an *Aphanomyces* resistance locus, encompassing an *IPT* candidate gene, correlated both with reduced disease symptoms and with the induction of lateral root formation in response to infection [64]. We could however not directly link the *cre1 Aphanomyces* tolerance phenotype with a differential colonization of primary *versus* lateral roots.

*cre1* roots are also resistant to salt stress, as previously observed in Arabidopsis *ahk* mutants at the seedling level [25], and this phenotype is also correlated with the ability of the *cre1* genotype to form an enhanced number of lateral roots even under salt stress conditions. We can then speculate that in both abiotic and biotic stress conditions, the increased mutant protection towards *A. euteiches* colonization and salt stress may be at least partially the consequence of the altered *cre1* root system architecture. A higher number of lateral meristems may indeed allow the plant to escape, at least transiently, from these stresses. Such basal/general defense mechanism, associated with a change in organ development, may additionally allow a simultaneous tolerance to different root pathogens [66]. This hypothesis does not exclude however that a direct crosstalk exists between the MtCRE1 cytokinin pathway and defense responses, as recently proposed in *Arabidopsis* shoots [30, 36], or that both direct and indirect effects are additive. Accordingly, the *Medicago cre1* mutant is unable to regulate in response to *Aphanomyces* inoculation the *RRA* gene Mt*RR4* as well as the key transcription factor Mt*EFD* (Ethylene Response Factor required For nodule Differentiation) controlling positively the *Ralstonia* pathogenic interaction [63].

Another interesting feature of the *cre1* mutant genotype is that it affects antagonistically pathogenic and symbiotic interactions. Crosstalks between symbiosis and immunity is an emerging topic, that relies notably on the strong structural similarities observed between plant LysM-domain containing receptor kinases which are able to recognize microbial chitosaccharide-derived molecules in both symbiotic or pathogenic interactions [24, 54]. A direct connection was recently demonstrated when mutants altered in the Nod Factor Perception (*NFP*) gene were shown to be more susceptible to *A. euteiches* than WT plants [67, 68]. In addition, the *M. truncatula EIN2/SICKLE (SKL)* pathway regulates negatively rhizobial (and mycorrhizal) symbiosis but positively defense against *Rhizoctonia solani* and *Phytophthora medicaginis* pathogens [69]. Finally, the EFD transcription factor regulates negatively symbiotic nodule initiation but positively the *Ralstonia solanacearum* pathogenic interaction [63]. All these data suggests a complex network of connections between symbiosis and immunity signaling. On one side, NFP and EFD are respectively positive and negative regulators of both symbiosis and defense; on the other side, the EIN2/SKL-ethylene and CRE1-cytokinin pathways regulate in an opposite direction the fate of each type of biotic interaction. In each case, the phenotypes may result from a combination between direct interactions with defense response pathways and/or indirect consequences of an altered root system development.

By testing in a single study various environmental factors, we could identify that the CRE1-dependent cytokinin pathway was integrating several root developmental and environmental responses, even though mechanisms underlying these different functions and their potential interactions remain to be determined. Whereas mutation of the *M. truncatula CRE1* pathway does not interfere negatively with the mycorrhizal symbiotic interaction, it affects root system architecture and allows an increased resistance to different environmental stresses, such as salt and the root pathogens *Aphanomyces* (this study) and *Ralstonia* [63]. We previously showed that, even though the *cre1* mutants form a lower nodule number, these organs are multilobed and bigger than in the WT [18]. Consequently, *cre1* mutant plants are able to fix nitrogen with a similar efficiency as WT plants (S3 Fig.). Affecting specific genes involved in cytokinin pathways may therefore be an interesting agronomical target to generate a basal root resistance to several environmental stresses without affecting beneficial endosymbioses.

## Supporting Information

S1 Fig *cre1* mutants do not have major meristem defects. (PDF)

S2 Fig Heat map of *M. truncatula* cytokinin metabolic gene expression in roots exposed to various environmental conditions. (PDF)

S3 Fig *cre1* mutants have a nitrogen fixation capacity similar to Wild-Type plants. (PDF)

S1 Table Primer list. (PDF)

S2 Table Gene IDs and names. (XLS)
