# Supplementary material for: The CRE1 Cytokinin Pathway Is Differentially Recruited Depending on Medicago truncatula Root Environments and Negatively Regulates Resistance to a Pathogen
Source: PLoS One. 2015 Jan 6;10(1):e0116819. doi: 10.1371/journal.pone.0116819 (PMC4285552; doi:10.1371/journal.pone.0116819)
Supplement: S1 Fig — (PDF) [file pone.0116819.s001.pdf]

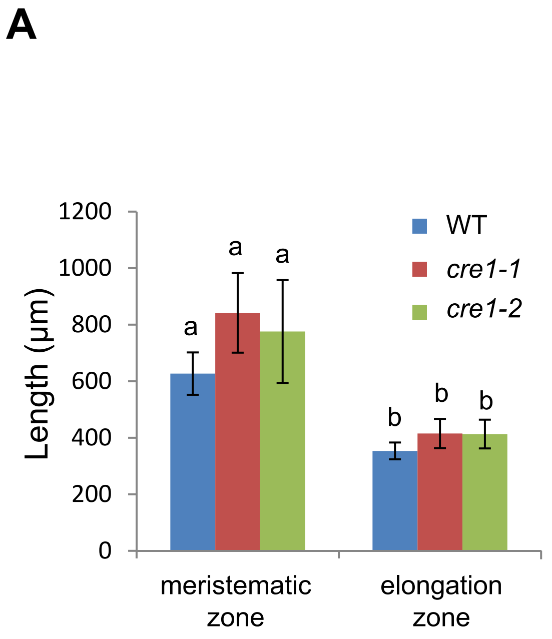

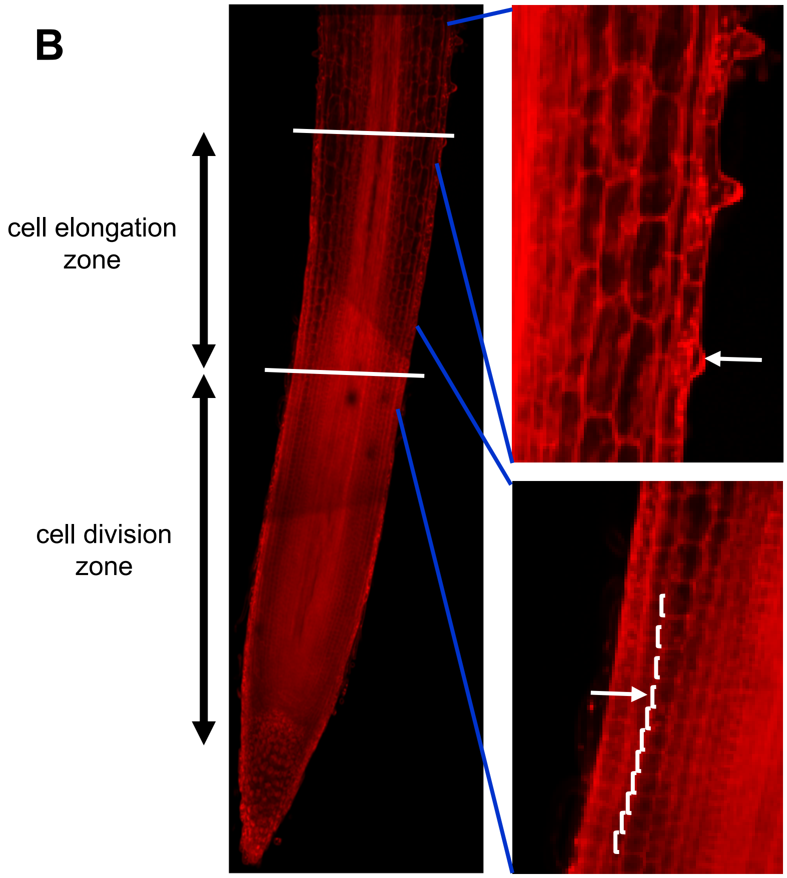

Figure S1. *cre1* mutants do not have major meristem defects
A. Length of root meristematic cell division and elongation zones of the Wild-Type (WT) and of the *cre1* mutant (*cre1-1* and *cre1-2* alleles). Error bars represent standard deviation. A Kruskal-Wallis test was performed ($\alpha < 5$ %; n = 10 roots), and the letters indicate significant differences.
B. Measurements in (**A**) were realized on roots stained with Propidium Iodide / Periodic Acid (PI-PA) and visualized under a confocal microscope. Zones were defined as follows (based on the outer cortical cell layer): the cell division zone from the quiescent center to the first elongated cell (arrow in lower inset; white brackets allow to visualize size of meristematic cells); and the elongation zone till the first differentiated root hair (arrow in upper inset).
