# Supplementary material for: The CRE1 Cytokinin Pathway Is Differentially Recruited Depending on Medicago truncatula Root Environments and Negatively Regulates Resistance to a Pathogen
Source: PLoS One. 2015 Jan 6;10(1):e0116819. doi: 10.1371/journal.pone.0116819 (PMC4285552; doi:10.1371/journal.pone.0116819)
Supplement: S2 Fig — (PDF) [file pone.0116819.s002.pdf]

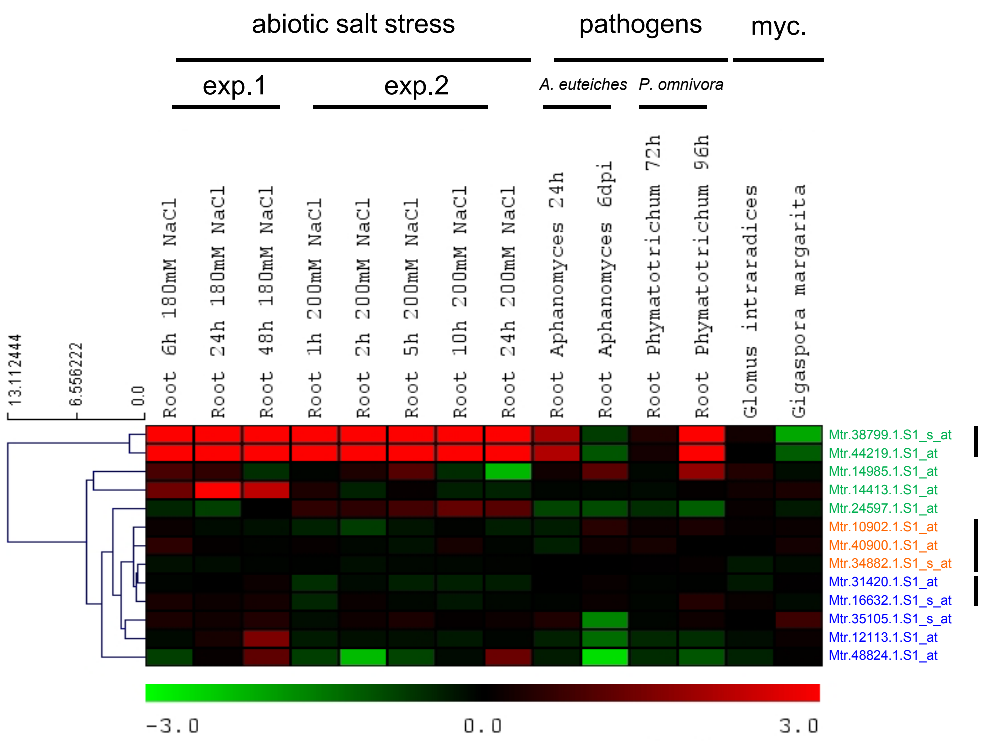

Figure S2. Heat map of *Medicago truncatula* cytokinin metabolic gene expression in roots exposed to various environmental conditions

Selected Affymetrix array data corresponding to roots under various abiotic and biotic conditions were retrieved from the *M. truncatula* Gene Expression Atlas (MtGEA) database: "myc.", mycorrhized roots (Gomez et al., 2009 and Ortu et al., 2012); salt stress, two independent experiments (exp. 1 and exp. 2; Li et al., 2009); *Phymatotrichopsis omnivora* and *Aphanomyces euteiches* pathogens (respectively Uppalapati et al., 2009 and Rey et al., 2013). All probes corresponding to cytokinin metabolic genes were included in the heat map, which was constructed with logarithmic gene expression ratio between the different conditions and their respective controls, based on Euclidean distance and average clustering of probes across the experimental conditions included, using the MeV software. Color scale ranges from eight time fold-repression in green (log2=-3) to eight time fold-induction in red (log2=3). Accession numbers correspond to Affymetrix probes (correspondence with gene ID in Table S2), and multiple probes corresponding to a single gene are indicated by a vertical black bar on the right. Colors indicate cytokinin metabolic gene families: in blue, IPTs (Iso PentenylTransferase); in orange, LOG (Lonely Guy cytokinin activating enzyme); in green, CKXs (Cytokinin Oxydase / Deshydrogenase).
