# Supplementary material for: The CRE1 Cytokinin Pathway Is Differentially Recruited Depending on Medicago truncatula Root Environments and Negatively Regulates Resistance to a Pathogen
Source: PLoS One. 2015 Jan 6;10(1):e0116819. doi: 10.1371/journal.pone.0116819 (PMC4285552; doi:10.1371/journal.pone.0116819)
Supplement: S3 Fig — (PDF) [file pone.0116819.s003.pdf]

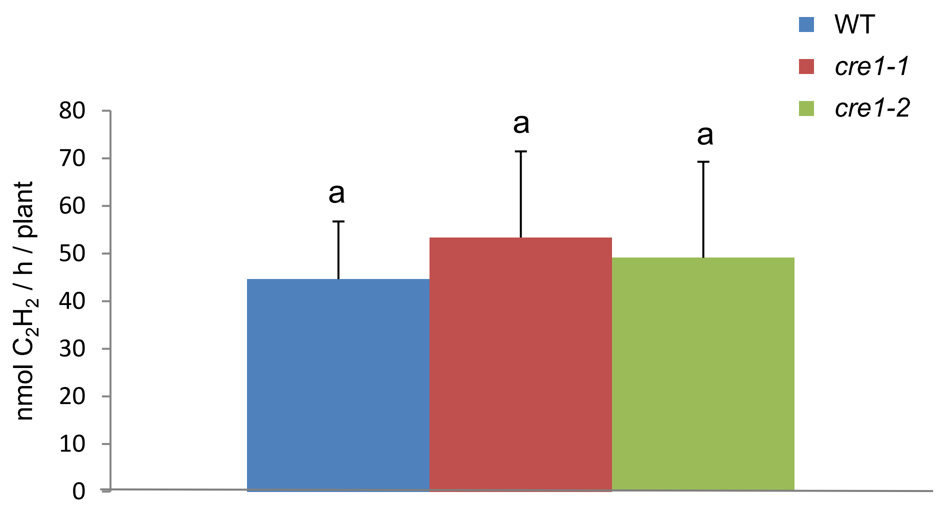

Figure S3. ***cre1*** **mutants have a nitrogen fixation capacity similar to Wild-Type plants**

Nitrogen fixation activity of Wild-Type (WT) and *cre1* plants (*cre1-1* and *cre1-2* alleles) five weeks post-inoculation with Rhizobium was determined using an Acetylene Reduction Assay (ARA). A Kruskal-Wallis test was performed ($\alpha < 5$ %; n = 10), and the letters indicate significant differences.
