# Supplementary material for: The CRE1 Cytokinin Pathway Is Differentially Recruited Depending on Medicago truncatula Root Environments and Negatively Regulates Resistance to a Pathogen
Source: PLoS One. 2015 Jan 6;10(1):e0116819. doi: 10.1371/journal.pone.0116819 (PMC4285552; doi:10.1371/journal.pone.0116819)
Supplement: S1 Table — (PDF) [file pone.0116819.s004.pdf]

Table S1. Primer list

H3L F	ATTCCAAAGGCGGCTGCATA
H3L R	CTTTGCTTGGTGCTGTTTAGATGG
RBP1 F	AGGGGCAAGTTCCTTCATTT
RBP1 R	GGTAGAAGTGCTGGCTCAGG
CRE1 F	CACCACCCTTTGGCTTCTAA
CRE1 R	CACTAAGTAGCGGCCTTTCG
CHK2 F	AGTCAACCACGGTTTTCCTC
CHK2 R	TCCTCCCAACAAGAAGATGG
CHK3 F	GGGTACATCAATTGGCATCC
CHK3 R	TCGTGCGAAACAGTAGCAAG
CHK4 F	AATCCGGGCTGAGGTTACAC
CHK4 R	CGCCATTCTGTCCTTGTTTC
